# Influenza A virus-mediated priming enhances cytokine secretion by human dendritic cells infected with *Streptococcus pneumoniae*

**DOI:** 10.1111/cmi.12122

**Published:** 2013-03-14

**Authors:** Thomas Kuri, Anna Smed Sörensen, Saskia Thomas, Gunilla B Karlsson Hedestam, Staffan Normark, Birgitta Henriques-Normark, Gerald M McInerney, Laura Plant

**Affiliations:** 1 Department of Microbiology, Tumor and Cell Biology, Karolinska InstitutetNobels väg 16, Stockholm, SE-171 77, Sweden; 2 Department of Laboratory Medicine Division of Clinical Microbiology, Karolinska University HospitalStockholm, SE-171 76, Sweden

## Abstract

Secondary infections with *Streptococcus pneumoniae* (SP) are frequently observed following influenza A virus (IAV) infection and have a substantial impact on global health. Despite this, the basis for the disease progression is incompletely understood. To investigate the effect of co-infection on human monocyte-derived dendritic cells (MDDCs) we analysed the expression of clinically important pro-inflammatory and immune-modulatory cytokines. IAV infection or treatment with supernatants from IAV-infected cell cultures resulted in priming of the DCs which subsequently influenced the production of IL-12p70, as well as IL-6, following SP infection. Co-infection of the same cell was not required but this effect was dependent on the time, dose and duration of the infections, as well as pathogen viability, bacterial uptake and endosome acidification. Bacterially infected cells were characterized as the main producers of IL-12p70. Finally, we showed that type I interferons were primarily responsible for the priming of IL-12p70 that was observed by infection with IAV. These results provide a probable mechanism for the elevated levels of particular cytokines observed in IAV and SP co-infected cell cultures with implications for the pathogenic outcome observed during *in vivo* infection.

## Introduction

Influenza A virus (IAV) and the bacterium *Streptococcus pneumoniae* (SP) are major human respiratory tract pathogens. Both are responsible for significant morbidity and mortality worldwide and constitute a critical issue for global health. Pneumococcal infections account for 1–2 million deaths annually and are the major cause of community-acquired pneumonia as well as more severe invasive diseases including septicaemia and meningitis (McCullers, 2006). IAV has caused approximately 30 pandemics over the past 400 years and infects millions of humans every season (Viboud *et al*., 2010). However, despite the severity of both pathogens individually, the most devastating outcomes occur if IAV and SP infect concurrently or in close temporal proximity. Current data indicate that co-infections have been responsible for millions of fatalities during the severe influenza pandemic in 1918–19 (McCullers, 2006; Morens *et al*., 2008). This finding is also supported by studies of patients from seasonal outbreaks (Tasher *et al*., 2011), as well during the IAV pandemic in 2009, in which severe illness correlated with secondary infections predominantly caused by SP (Palacios *et al*., 2009; Viasus *et al*., 2011). IAV infections in combination with secondary bacterial infections have been found to be one of the leading causes of death in the USA (Kochanek *et al*., 2011) highlighting the need for a better understanding of co-infections. Despite epidemiological data indicating that a substantial proportion of influenza-related deaths are attributable to secondary bacterial infections the basis for the pathogenic outcome of co-infections is unclear.

Neutrophils and alveolar macrophages predominate during the initial innate immune response to SP; however, adaptive immune mechanisms are crucial for bacterial clearance and host responses upon infection (Alonso De Velasco *et al*., 1995). A connection between an initial innate immune reaction and a pathogen-specific adaptive immune response is represented by the action of dendritic cells (DCs). On the other hand, viruses have developed several mechanisms to interfere with the function of DCs and prevent the normal development of adaptive immunity (Salio *et al*., 1999; Rodriguez-Madoz *et al*., 2010; Hansen *et al*., 2011). Much of the current knowledge regarding viral/bacterial co-infections has been obtained by studying co-infection in mice where IAV infection alters the expression and exposure of bacterial receptors (McCullers and Rehg, 2002; McCullers and Bartmess, 2003) and affects respiratory tract cell viability which promotes bacterial spread (Kosai *et al*., 2011). Infection with IAV has also been shown to cause a systemic suppression of the immune response which, in turn, supports secondary bacterial infections (Jamieson *et al*., 2010). Furthermore, virus-induced modulations of cytokine production that affect host responses to SP were described (van der Sluijs *et al*., 2004; Kukavica-Ibrulj *et al*., 2009; Zavitz *et al*., 2010; Kudva *et al*., 2011). In this context, type I and II interferon (IFN) can be disadvantageous for the infected host during subsequent bacterial infection (Sun and Metzger, 2008; Shahangian *et al*., 2009; Nakamura *et al*., 2011; Li *et al*., 2012). Despite the usefulness of mouse models for studying human infections, differences in human and murine DCs, for example in their capacity to produce IL-12p70 and IL-1β in response to pneumococcal infection (Littmann *et al*., 2009), highlight the need for more research on human systems to study the effect of this viral/bacterial co-infection. Recently, elevated cytokine levels in IAV-infected cells after co-infection with SP were reported in human monocyte-derived DCs (MDDCs) stimulated with killed bacteria (Wu *et al*., 2011). A recent patient study also described elevated levels of IL-12p70, IL-6 and IL-15 as markers for severe clinical outcome after IAV infection (Bermejo-Martin *et al*., 2009). The regulation of the production of cytokines and chemokines is crucial for the infected host to launch appropriate, non-harmful immune responses. The composition of the host cytokine response strongly influences the nature of adaptive immune responses, e.g. IL-4 promotes T-helper (Th) cell polarization into Th2 responses and IL-12p70 promotes Th1, while IL-6 supports Th17 and Th22 responses (Dong, 2008; Annunziato and Romagnani, 2009). DCs play roles in these processes by production of cytokines and presentation of antigens to cells of the adaptive immune system, hence DCs bridge the innate and the adaptive arms of the immune response (Pulendran *et al*., 2001; Iwasaki and Medzhitov, 2004; Volz *et al*., 2012).

In this study we developed a human-cell-based co-infection system using viable IAV and SP in order to examine the impact of the virus infection on DC function and the consequences of a subsequent infection with SP. We found that IAV infection of MDDC cultures significantly enhanced the induction of IL-12p70, as well as IL-6, following SP infection. Co-infection of the same cell was not required for enhanced cytokine secretion as the main IL-12p70 producers were infected with bacteria alone. Furthermore, we found that pre-treatment of MDDCs with interferon-α similarly enhanced IL-12p70, as well as IL-6, production following SP infection. We determined that the priming effect of IAV on SP induction of IL-12p70 was dependent on regulation of the p35 subunit of IL-12p70 since treatment with IAV and SP led to enhanced induction of expression of the p35 subunit, with no effect observed on the p40 subunit. Neutralizing type I IFN antibodies abrogated the induction of the IL-12p35 subunit confirming that IAV-induction of type I IFNs was the main pathway by which IAV primed MDDCs for enhanced IL-12p70 production following SP infection.

## Results

### Co-infection of MDDCs leads to elevated levels of IL-12p70 and IL-6

**Fig 1:**
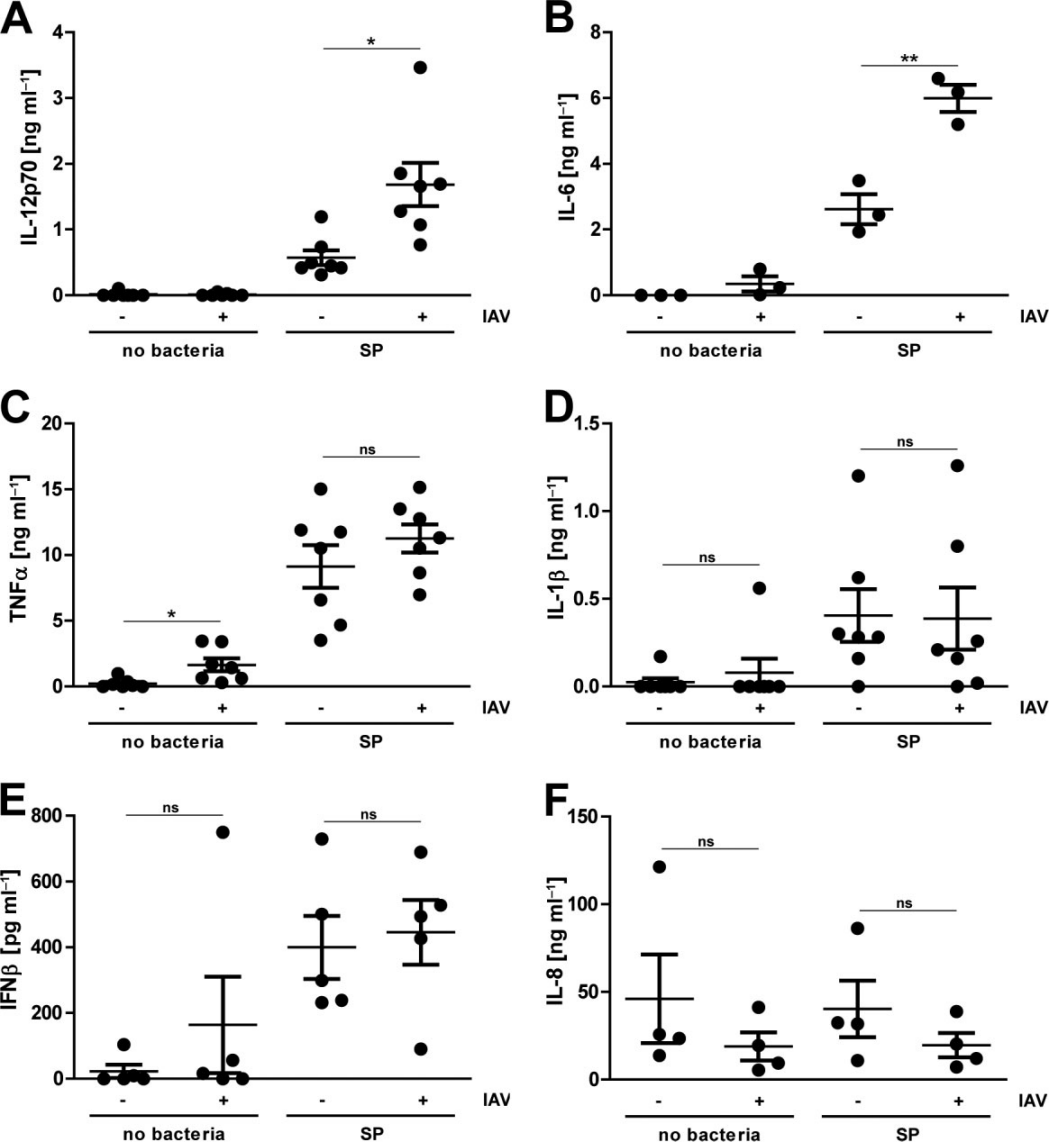
Preceding influenza infection leads to elevated levels of secreted IL-12p70 and IL-6 after co-infection with SP. MDDCs were infected with IAV for 4 h before infection with SP. The cells were further incubated for 18 h before the concentration of IL-12p70 (A), IL-6 (B), TNFα (C), IL-1β (D), IFN-β (E) and IL-8 (F) in the supernatants was measured by ELISA. Each dot in a graph represents the cytokine concentration from cells of one donor. The graphs show mean ± SEM of seven (A, C), three (B), five (D, E) or four (F) independent experiments with different donors. Statistical analysis was performed using paired Student’s *t*-test. (*P < 0.05, **P < 0.01).

We established a co-infection system involving human MDDCs, replication-competent virus and live bacteria and have used it to characterize the effect of IAV and SP co-infections on key antigen-presenting cells. The levels of various secreted antiviral, pro-inflammatory and immune-modulatory cytokines and chemokines from MDDCs were determined by ELISA to examine potential changes in expression after co-infection. We found significantly elevated levels of IL-12p70 and IL-6 in IAV-infected MDDCs exposed to SP, while infection with either pathogen alone induced low or undetectable levels of both cytokines (Fig. 1A and B). Other cytokines including TNFα, IL-1β and IFN-β were also induced by IAV and SP; however, no significant change in expression was found following co-infection (Fig. 1C–E). Uninfected MDDCs produced substantial amounts of IL-8 which decreased after IAV infection. However, co-infection had no further impact on the levels of this chemokine (Fig. 1F). The levels of IL-10 and IL-15 were also measured but no detectable amounts of these cytokines were recorded in response to either pathogen alone or in combination (data not shown).

**Fig 2:**
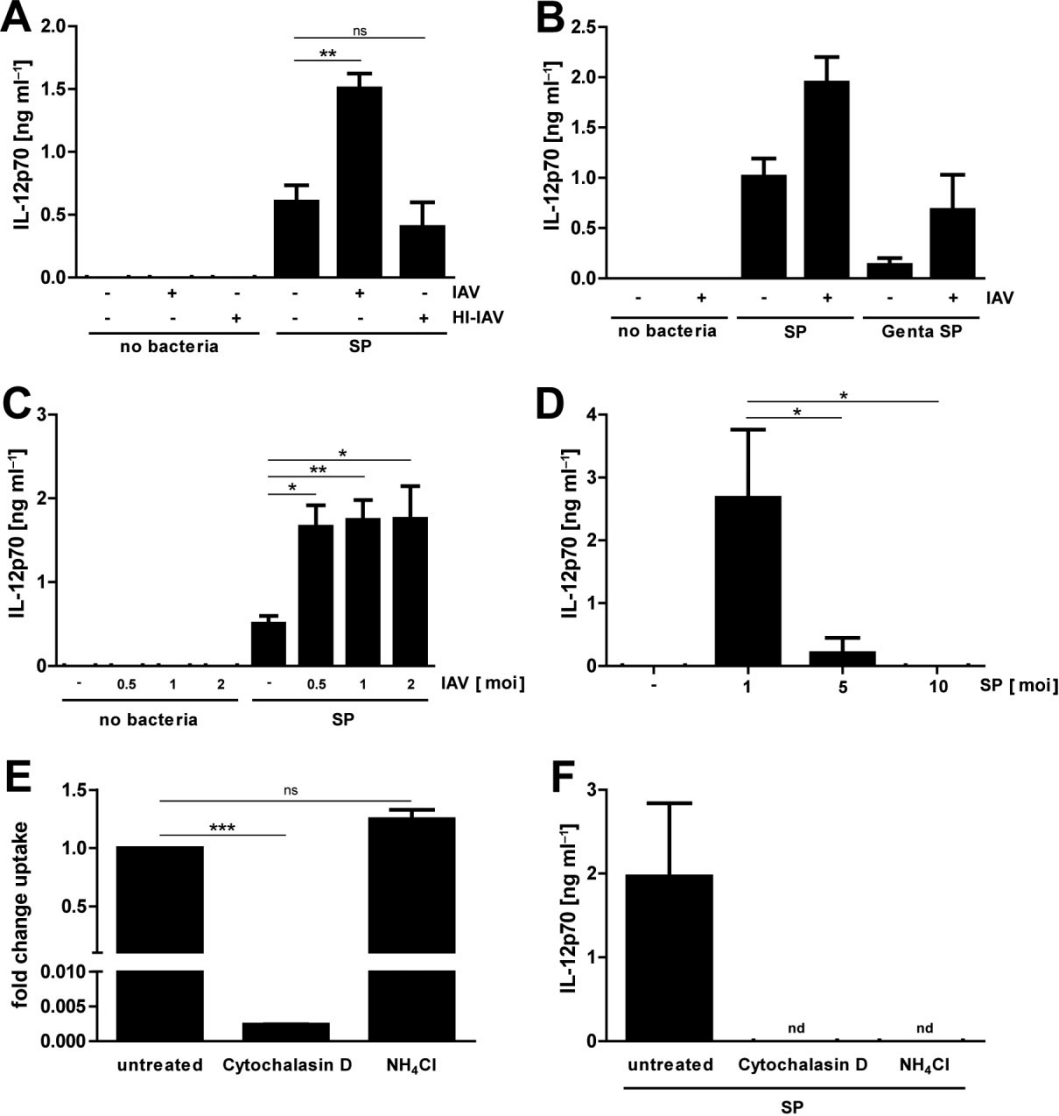
IL-12p70 secretion depends on bacterial uptake as well as dose and viability of both pathogens. MDDCs were infected with IAV for 4 h before infection with SP. The cells were further incubated for 18 h before the concentration of IL-12p70 in the supernatants was determined. Different infection conditions were tested for their potential to induce an elevated cytokine response.
IAV/heat-inactivated (HI) IAV moi 0.5 and SP moi 1IAV moi 0.5 and viable or gentamicin-killed SP moi 1.Increasing moi of IAV and SP moi 1.Increasing moi of SP. MDDCs were incubated with Cytochalasin D or NH_4_Cl 15 min before infection with SP.The number of intracellular, viable SP was determined by culturing serial dilutions of cell lysates and normalized against the uptake rates of uninfected control cells.Cytochalasin D-and NH_4_Cl-treated MDDCs were infected with SP and incubated for 18 h before the concentration of IL-12p70 in the supernatants was determined by ELISA. Values represent mean ± SEM of six (A), two (B), four (C), seven (D) or three (E, F) independent experiments with different donors. Statistical analysis was performed using paired Student’s *t*-test. (*P < 0.05, **P < 0.01, ***P < 0.001); nd, not detectable.

As IAV infection alone did not lead to secretion of biologically active, Th1-polarizing, IL-12p70 but instead caused a priming of MDDCs, we chose to analyse the effect on this cytokine in greater detail. To determine the requirements for the enhanced cytokine induction, the impact of bacterial uptake and processing, as well as different moi and viability of the pathogens were tested for their ability to induce IL-12p70 (Fig. 2). Stimulation with heat-inactivated virus had no effect on cytokine levels, showing that viral replication is needed for priming of the induction of IL-12p70 (Fig. 2A). The level of cytokine produced was also dependent on the bacterial viability since heat-killed SP (data not shown) and gentamicin-killed SP (Fig. 2B) induced only low amounts of IL-12p70. Despite the decreased induction of IL-12p70 by gentamicin-killed SP, a similar trend was observed with a priming effect observed in the context of a co-infection. Elevated IL-12p70 levels were detected for all doses of virus that were tested (Fig. 2C). The highest levels of IL-12p70 were found following infection with a bacterial moi of 1 and the levels of IL-12p70 decreased significantly with increasing moi (Fig. 2D). To examine the impact of bacterial uptake and processing of bacteria on the induction of IL-12p70 we used cytochalasin D to disrupt the actin filaments and thereby inhibit phagocytic activity of the MDDCs, as well as NH_4_Cl to prevent endosome acidification. Treatment of MDDCs with cytochalasin D strongly reduced the number of intracellular bacteria, whereas blocking endosome acidification had no effect on bacterial uptake (Fig. 2E). However, both treatments completely abrogated the induction of IL-12p70 (Fig. 2F) while the general capacity of the cells to produce IL-12p70 was not affected (data not shown). To exclude the possibility of enhanced cell death due to the different infection conditions and treatments, the viability of the cells for all conditions was monitored by measuring lactate dehydrogenase (LDH) release. No increased cell death was detected for the tested conditions (data not shown).

**Fig 3:**
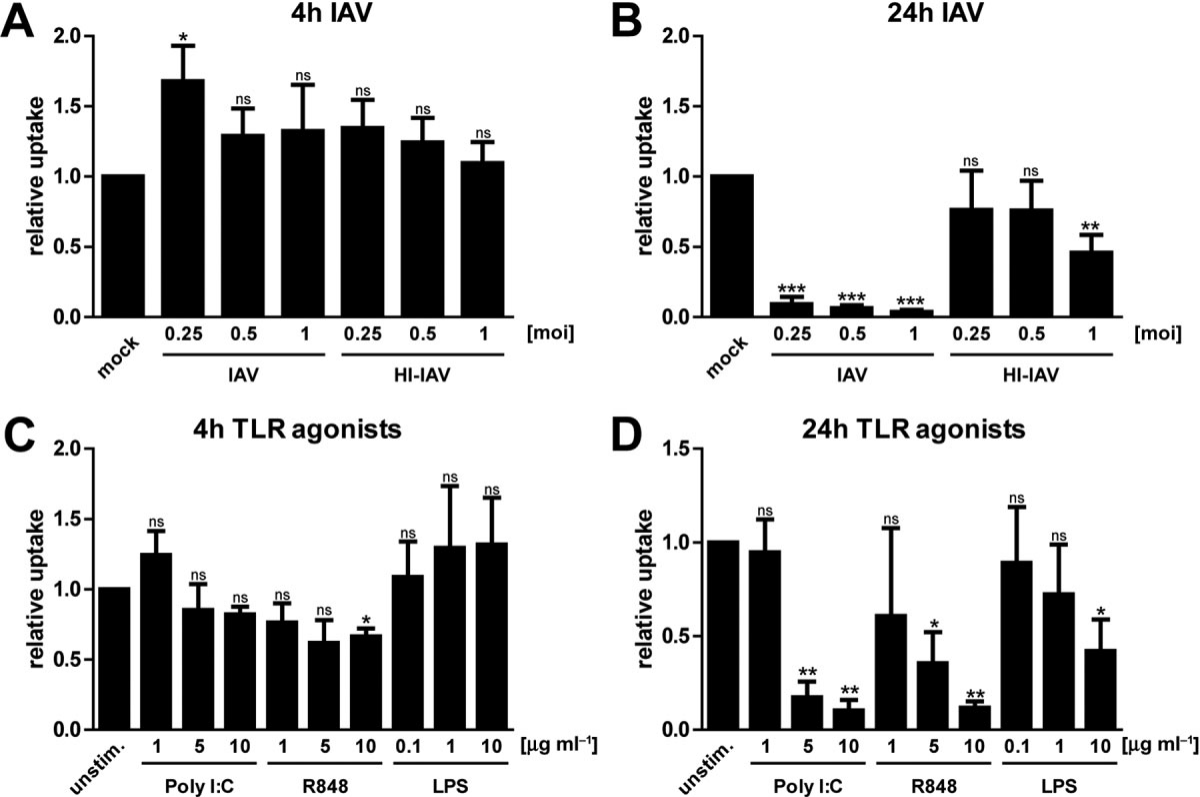
Influence of IAV infection on bacterial uptake by MDDCs. MDDCs were infected with IAV for 4 h (A) and 24 h (B) or stimulated with TLR agonists PolyI:C, R848, LPS for 4 h (C) or 24 h (D) before SP was added. The number of intracellular, viable SP was determined by culturing serial dilutions of cell lysates and normalized against the uptake rates of uninfected control cells. Values represent mean ± SEM of six independent experiments with different donors. Statistical analysis was performed using paired Student’s *t*-test. (*P < 0.05, **P < 0.01, ***P < 0.001).

The duration of IAV infection strongly affected the observed priming of MDDCs. Cells that were infected with IAV for 24 h produced less IL-12p70 compared with MDDCs infected for 4 h prior to exposure to SP (data not shown). Hence, bacterial uptake, processing of the internalized bacteria and the number and viability of SP determined whether IL-12p70 was produced by MDDCs, while a priming of IL-12p70 induction was only found after infection with replication-competent IAV. A similar IL-6 response was observed following co-infection, although the increase appeared to be additive rather than synergistic for some donors since virus infection alone already induced secretion of robust amounts of this cytokine (Fig. S1). Interestingly, in contrast to IL-12p70 the amount of secreted IL-6 increased with higher bacterial moi.

### Infection with IAV decreases uptake of pneumococci at late infection time points

We investigated whether IAV infection would affect the rate at which SP is taken up by MDDCs which might, in turn, influence their response towards the bacteria. As priming of the induction of IL-12p70 was dependent on the duration of IAV infection, time points at 4 h and 24 h post infection were chosen to study bacterial uptake (Fig. 3A and B). At 4 h post infection a slightly higher uptake rate was found if the cells had been infected with IAV at moi 0.25 but no significant changes were found for higher moi or stimulation with heat-inactivated IAV for 4 h even though the cytokine expression at the same condition was markedly different. Furthermore, time-course experiments revealed that the clearance of bacteria at early time points after infection was not influenced by IAV, as MDDCs in co-infected cultures killed the bacteria with the same kinetics as MDDCs that were infected with SP alone. Independent of the infection with IAV, all MDDCs cleared the phagocytosed bacteria in less than 5 h after uptake (Fig. S2A). After 24 h of IAV infection the uptake of SP decreased strongly in a dose-dependent manner. Infection with viable virus was superior in the capacity to decrease SP uptake by MDDCs compared with exposure with heat-inactivated virus which affected uptake only at the highest moi tested. In order to determine whether this effect was specific for IAV, MDDCs were treated with agonists for the Toll-like receptors (TLR) 3, TLR4 and TLR7/8 (Poly I:C, LPS, R848 respectively) for either 4 h or 24 h prior to infection with SP (Fig. 3C and D). While stimulation for 4 h had no effect on uptake rates except for the highest concentration of R848, 24 h of stimulation led to a dose-dependent change in uptake of bacteria for all applied TLR agonists with increasing ligand concentrations decreasing bacterial uptake. The same trend was found for MDDCs infected with the non-related Semliki Forest virus (Fig. S2B). In this case, similar to stimulation with TLR agonists, bacterial uptake decreased in a dose-dependent manner after 24 h but not after 4 h post infection. Taken together these results indicate that the observed change in phagocytosis of SP is not a specific consequence of an infection with IAV but likely reflects an activation of innate immune pathways in MDDCs which, in turn, results in decreased bacterial uptake over time. These findings are in line with other reports showing that prolonged stimulation decreases the phagocytic capacity of DCs (West *et al*., 2000; Spadaro *et al*., 2012).

### TLR agonists enhance the SP-dependent production of IL-12p70 by MDDCs

**Fig 4:**
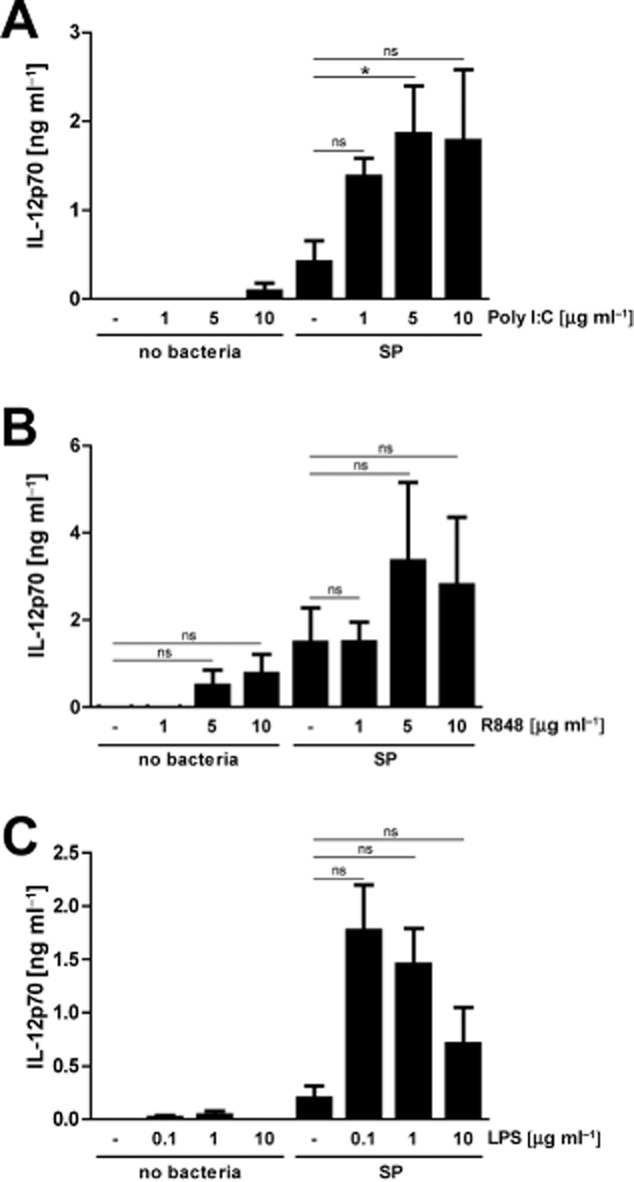
Priming of the induction of IL-12p70 secretion can be stimulated by a combination of TLR agonists and SP. Different doses of TLR3 (A), TLR7/8 (B) or TLR4 agonists (C) were applied for 4 h before SP was added. The cells were incubated for another 18 h and the concentration of IL-12p70 in supernatants was determined by ELISA. Values represent mean ± SEM of five independent experiments with different donors. Statistical analysis was performed using paired Student’s *t*-test. (*P < 0.05).

The production of IL-12p70 by MDDCs can be induced by various individual TLR ligands; however, a simultaneous stimulation with selected TLR ligands has been shown to prime the induction of this cytokine, since MDDCs are able to integrate signals from different TLRs (Napolitani *et al*., 2005; Bohnenkamp *et al*., 2007). Similarly, we observed that stimulation with a combination of a TLR7/8 ligand (R848) and a TLR4 ligand (LPS) led to increased production of IL-12p70 by MDDCs (data not shown). Since the various TLR agonists and IAV influenced bacterial uptake to a similar extent we investigated whether single TLR ligands in combination with bacterial infection would result in elevated levels of IL-12p70. Therefore, MDDCs were stimulated with TLR3, TLR4 or TLR7/8 agonists before the cells were infected with SP. The addition of Poly I:C (Fig. 4A), R848 (Fig. 4B) or LPS (Fig. 4C) increased the amount of IL-12p70 induced by pneumococcal infection, while the TLR agonists alone induced lower levels of IL-12p70. However, although large differences were observed in all donors, the average differences observed upon compilation of donors were mostly non-significant. Likewise, we also found elevated levels of IL-6 after a combined stimulation of TLR agonists and SP (Fig. S3). Despite the robust secretion of IL-6 after stimulation with single TLR ligands, their combination with bacteria led to higher levels. In line with findings following the infection with IAV, IL-12p70 levels did not increase when MDDCs were stimulated with the TLR ligands for 24 h before infection with SP (data not shown).

### IAV priming of DCs to produce IL-12p70 is facilitated by specific induction of the IL-12p35 subunit

**Fig 5:**
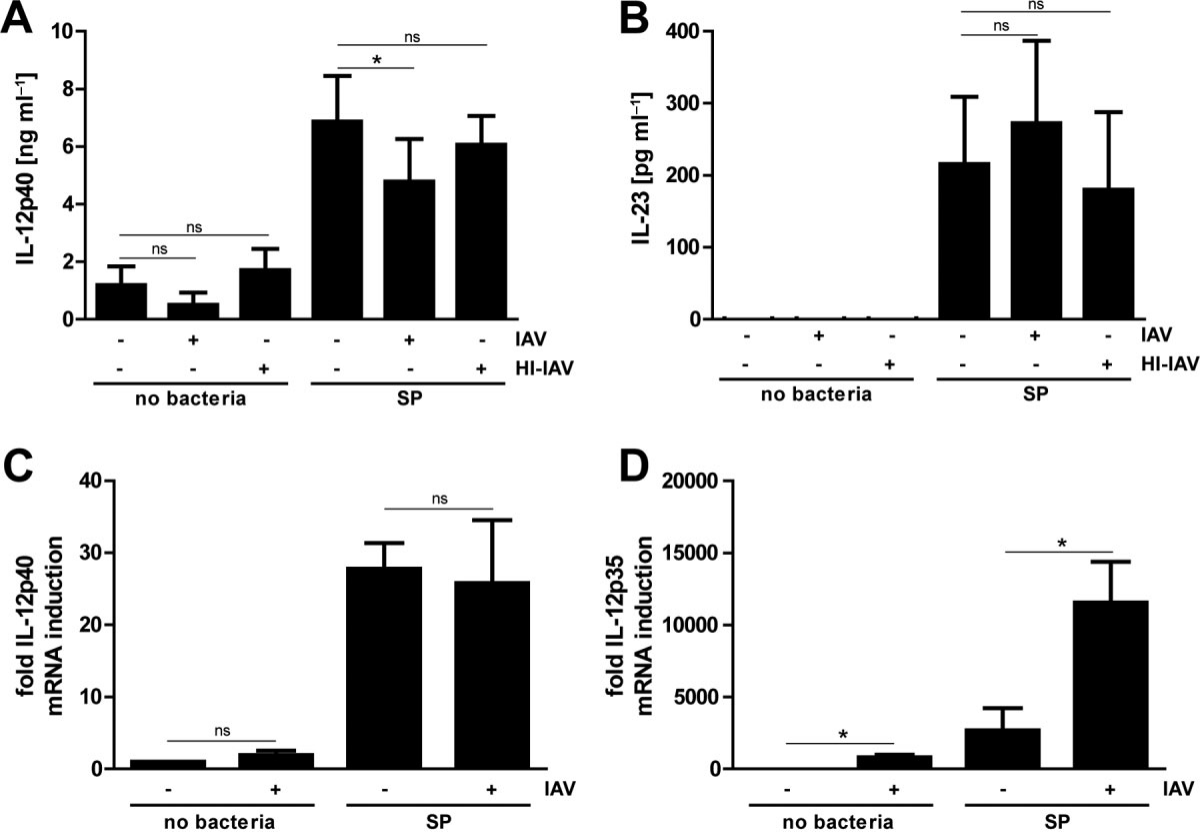
Enhanced IL-12p70 secretion is due to a specific induction of the IL-12p35 subunit. MDDCs were infected with IAV for 4 h before SP was added. The cells were then incubated for 18 h prior to monitoring the concentration of IL-12p40 (A) and IL-23 (B) in the supernatants by ELISA. Values represent mean ± SEM of five (A) or four (B) independent experiments with different donors. The transcription of the IL-12p40 (C) and IL-12p35 (D) subunits of IL-12p70 were monitored in MDDCs that were co-infected as described above. After 22 h incubation the cells were lysed and total RNA was isolated and assayed for the presence of IL-12p35 and IL-12p40 mRNA. Ct values were normalized against γ-actin and the relative induction of the genes was calculated using the ΔΔCt method. Values represent mean ± SEM of three independent experiments with different donors. Statistical analysis was performed using paired Student’s *t*-test. (*P < 0.05).

Biologically active IL-12p70 is secreted as a heterodimer consisting of a p35 and a p40 subunit. The p35 subunit is not secreted on its own and is a component of both IL-12p70 and the recently discovered cytokine IL-35, which is produced by regulatory T cells (Hamza *et al*., 2010). In addition to being a subunit of IL-12p70, IL-12p40 is a subunit of IL-23 and can be secreted as a monomer or a homodimer (Trinchieri, 2003). To assess the characteristics of the IAV priming of MDDCs to produce IL-12p70 after co-infection with SP the levels of IL-12p40 and IL-23 were examined (Fig. 5A and B). Interestingly, IL-12p40 and IL-23 levels were not enhanced by the co-infection compared with levels observed in response to SP alone. Rather, infection with IAV decreased the levels of IL-12p40. These data suggest that the IL-12p35 is a limiting factor for the observed synergism. To further investigate this hypothesis the mRNA levels of the two IL-12p70 subunits were determined by quantitative real-time PCR. In support of the ELISA data the level of IL-12p40 mRNA was not enhanced by co-infection with IAV and SP (Fig. 5C). However, the level of IL-12p35 mRNA was strongly increased when MDDCs were infected with both pathogens (Fig. 5D), suggesting that transcriptional induction of p35 mRNA leads to the enhanced IL-12p70 secretion observed after co-infection of MDDCs. Interestingly, a low but significant amount of IL-12p35 mRNA was detected after IAV infection although no IL-12p70 was detected in the supernatants.

### MDDCs infected with SP are the main producers of IL-12p70

**Fig 6:**
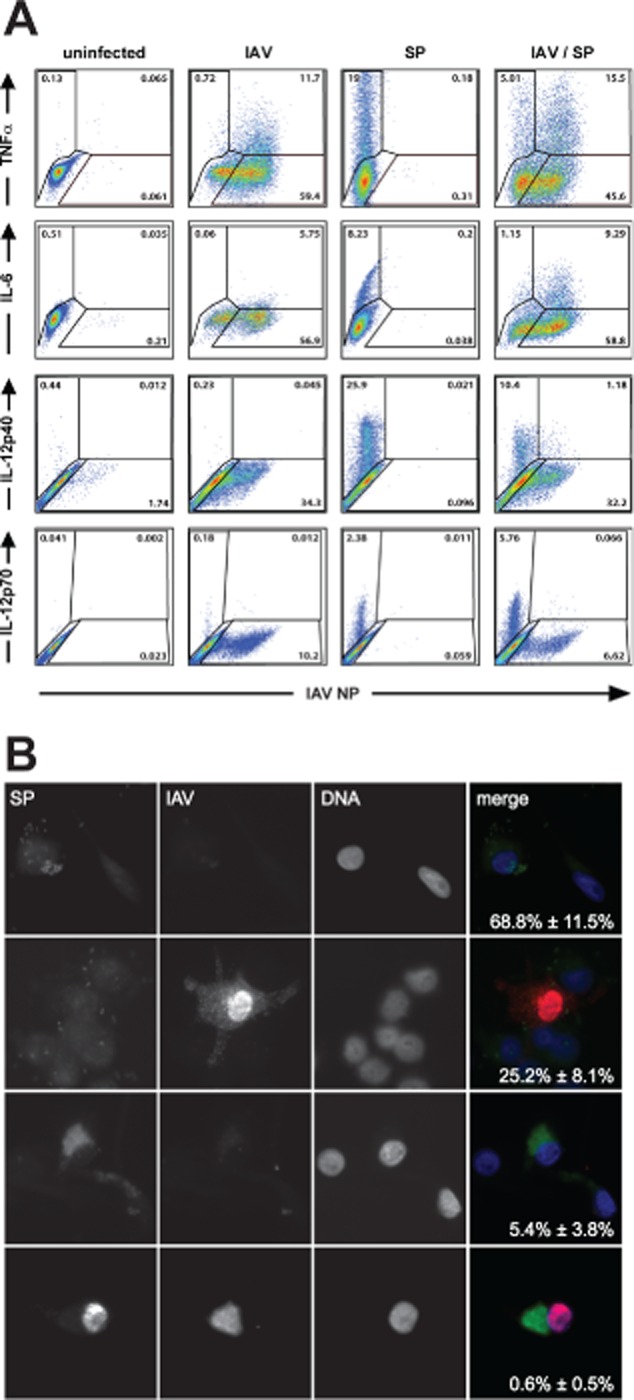
Intracellular cytokine staining and infection status of individual cells.
MDDCs were infected with IAV for 4 h before SP was added. After co-infection, brefeldin A or monensin was added to the cells to allow accumulation of intracellular cytokines. The cells were then fixed with paraformaldehyde, stained with specific antibodies and analysed for the presence of IAV nucleoprotein and TNFα, IL-6, IL-12p40 and IL-12p70 by FACS. The graphs show one representative donor of three.Infection status of single cells in co-infected cultures. MDDCs were seeded on glass slides and sequentially infected as described. The cells were fixed 4 h after addition of SP and stained with specific antibodies for SP (green), IAV (red) and Hoechst DNA stain (blue). Four hundred cells per donor were examined. Numbers in the right column show the average frequency of the observed pattern ± SD from three independent experiments with different donors.

In order to examine which cells were primarily responsible for the production of IL-12p70 in the MDDC co-infection system, we analysed the cytokine expression of the cells by flow cytometry using antibodies against IAV nucleoprotein (IAV NP) and specific cytokine antibodies (Fig. 6A). Infection with either pathogen resulted in expression of TNFα as well as IL-6, but SP induced more cytokine expression than the viral infection. IL-12p40 expression was not induced by IAV infection but required the infection with SP and co-infection with IAV resulted in decreased numbers of IL-12p40-positive cells. As expected, cells that were not exposed to bacteria did not produce IL-12p70, while approximately 2–6% of SP-infected cells were stained positive for IL-12p70. Interestingly, we found a clear dichotomy between IL-12p70-secreting and IAV-infected MDDCs as indicated by the staining of viral NP, showing that either the IL-12p70-producing cells were not infected with the virus or their level of viral nucleoprotein was below the detection limit. To further address this finding, MDDCs infected with IAV and SP were stained with antibodies against IAV NP and SP. Analysis by immunofluorescence microscopy revealed 4 distinct staining patterns in the co-infected MDDC cultures and quantification of those showed considerable differences regarding the frequency of the observed patterns (Fig. 6B). The majority of cells were found to be associated with single bacteria and negative for IAV infection (top row). Approximately 25% of all MDDCs were stained positive for IAV and were as well associated with single bacteria (second row). Around 6% were IAV-negative but showed a strong cytoplasmic staining positive for SP (third row) while no single bacteria could be detected in this population, indicating that these cells engulfed and had already digested SP. Very few cells were found to be double-positive for IAV and for digested SP (bottom row). Taken together, these results strongly suggest that the MDDCs which secreted IL-12p70 were exposed but not necessarily directly infected with IAV, but acquired and digested bacteria allowing innate immune receptors to sense infection and trigger the production of IL-12p70. This finding is further supported by the fact that MDDC infection with SP alone resulted in IL-12p70 secretion, while infection with IAV alone did not. A possible explanation for the enhanced cytokine secretion would be a virus-mediated stimulation of uptake and digestion of the bacteria that leads to a stronger response. However, no quantitative difference with respect to the number of MDDCs that had taken up and digested SP was found in the presence or absence of IAV-infected MDDCs at conditions where the enhanced cytokine secretion was observed (Fig. S4).

### IAV-infected MDDCs prime surrounding non-infected cells to promote an enhanced response towards a subsequent SP infection

**Fig 7:**
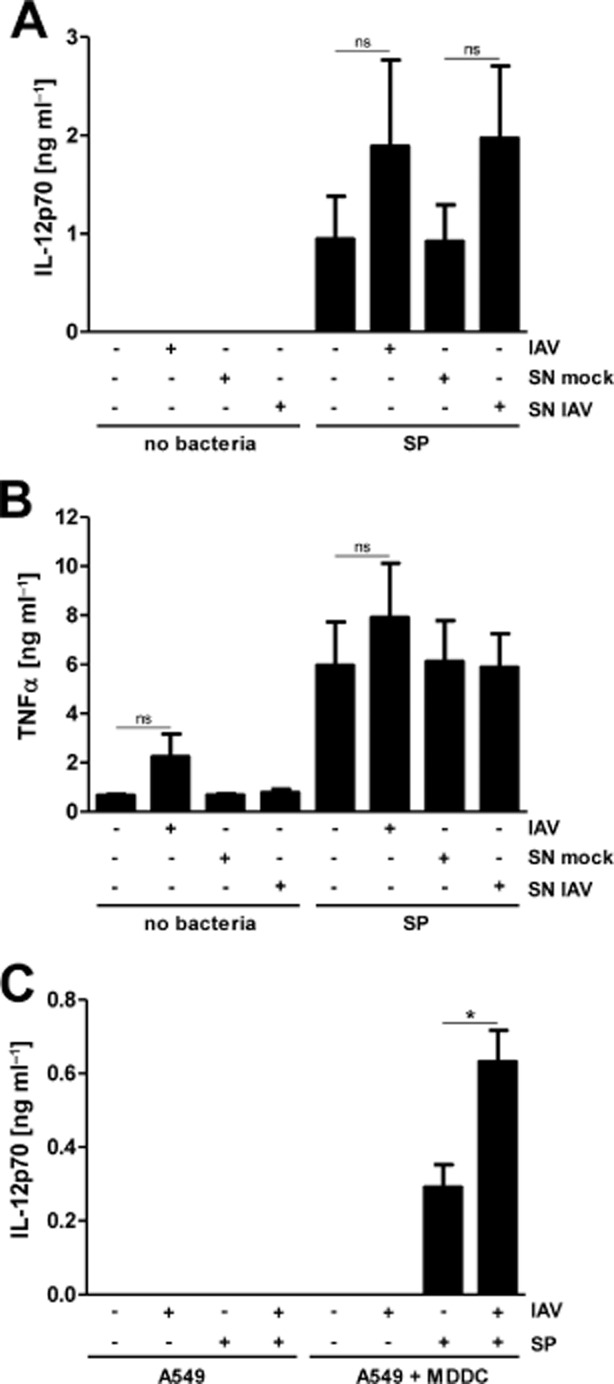
Supernatants of IAV-infected MDDCs prime non-infected cells to promote an enhanced IL-12p70 response to SP. MDDCs were infected with IAV or treated with supernatants of mock-infected (SN mock) or IAV-infected MDDCs (SN IAV) and incubated for 4 h.
The cells were then infected with SP and further incubated for 18 h before the concentration of (A) IL-12p70 and (B) TNFα in the supernatants was measured by ELISA.A549 cells were infected with IAV and incubated for 4 h. Then MDDCs were seeded in co-culture with the A549 cells and infected with SP. The co-cultures were incubated for further 18 h before the concentration of IL-12p70 in the supernatants was measured by ELISA. Values represent mean ± SEM of three (A, B) or five (C) independent experiments with different donors. Statistical analysis was performed using paired Student’s *t*-test. (*P < 0.05).

To determine whether the IAV-dependent priming of IL-12p70 expression after subsequent SP infection was mediated by a secreted factor, we performed supernatant transfer experiments where supernatants from mock-and IAV-infected cells were transferred to fresh MDDCs and the level of cytokine secretion was determined. The supernatants of mock-infected MDDCs did not alter the response of recipient MDDCs to the SP infection, yet supernatants of IAV-infected cells could prime MDDCs to produce IL-12p70 (Fig. 7A) in response to SP. A similar finding was observed for IL-6 (Fig. S5A). In line with our previous findings, the expression of TNFα and IL-12p40 was not enhanced when supernatant transfer experiments were performed (Fig. 7B and data not shown). Since only particular subsets of DCs allow productive infection with IAV (Moltedo *et al*., 2011), we considered it unlikely that the cytokine induction observed following the incubation with the supernatants was mediated by contaminating infectious IAV. Furthermore, newly produced IAV particles would require the proteolytic activation of the haemagglutinin for cell entry, but trypsin was absent in our cultures. However, to exclude the possibility of transferring infectious virus particles, the infection status of recipient cells was examined by immunofluorescence analysis and qRT-PCR. No viral infection could be detected, as judged by staining of IAV NP (Fig. S5B) or amplification of viral M1-mRNA (Fig. S5C). To further address the involvement of secreted factors, IAV-infected human epithelial lung cells (A549) were incubated in co-culture with SP-infected MDDCs (Fig. 7C). A549 cells alone did not produce any detectable IL-12p70 whether infected with virus and/or bacteria. However, MDDCs produced substantially more IL-12p70 in response to bacterial infection when they were kept in co-culture with IAV-infected A549 cells. These results show that the priming effect on these cytokines is not mediated by the IAV infection *per se* but rather by soluble factors induced by IAV and released from virus-infected cells.

**Fig 8:**
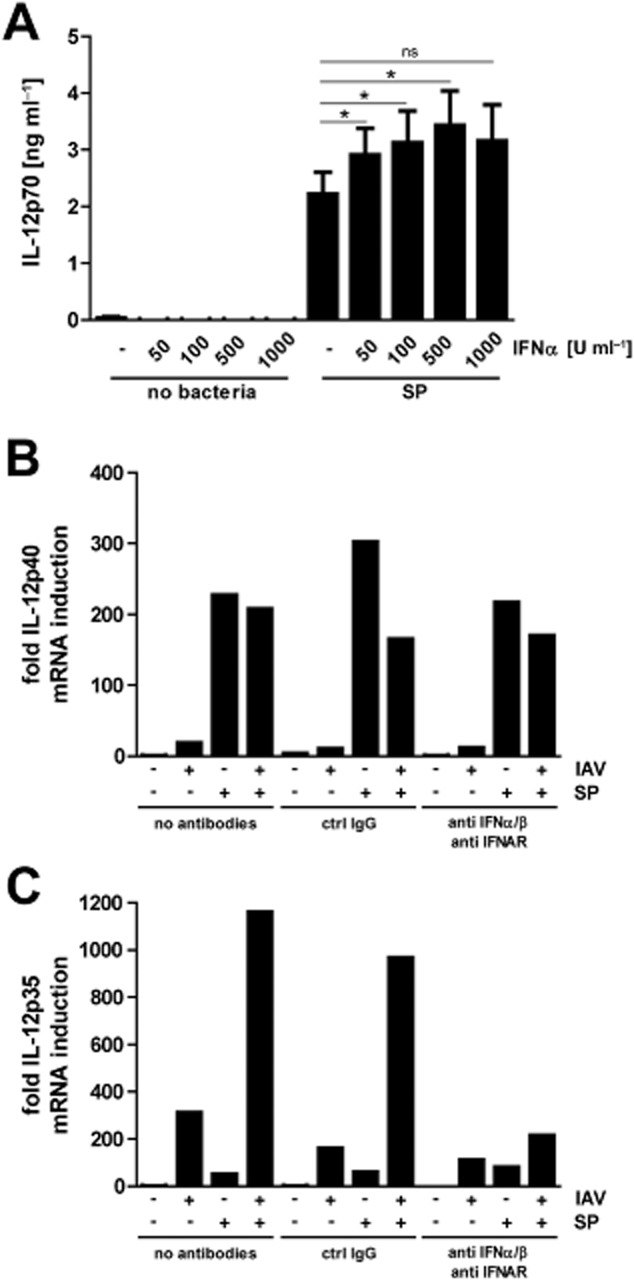
Type I IFNs are responsible for the priming of DCs to produce IL-12p70.
MDDCs were incubated with different concentrations of recombinant human IFN-α 4 h prior to infection with SP. The cells were further incubated for 18 h before the concentration of IL-12p70 in the supernatants was measured by ELISA.MDDCs were sequentially infected with IAV and SP in the presence of neutralizing antibodies against IFN-α, IFN-β and IFNAR. After 22 h incubation the cells were lysed and total RNA was isolated and assayed for the presence of IL-12p35 and IL-12p40 mRNA. Ct values were normalized against γ-actin and the relative induction of the genes was calculated using the ΔΔCt method. Values represent mean ± SEM three independent experiments with different donors (A) or one representative donor of 3 (B, C). Statistical analysis was performed using paired Student’s *t*-test. (*P < 0.05).

A hallmark of IAV infection is the induction of type I IFNs, which promote IL-12p70 responses induced by TLR agonists (Gautier *et al*., 2005). Therefore we asked whether type I IFNs mediated the observed priming effect. MDDCs were incubated with different concentrations of recombinant human IFN-α for 4 h before the cells were infected with SP. Strikingly, induction for IL-12p70 (Fig. 8A) was observed in a dose-dependent manner while the highest levels of cytokine was found after priming with an IFN-α concentration of 500 U ml^−1^. Similar findings were observed for IL-6 (Fig. S6). In parallel MDDCs of the same donors were co-infected with IAV and SP to compare the stimulation with recombinant IFN to an actual IAV infection. Here we found that the virus infection resulted in a stronger induction of the cytokines than priming with IFN-α (data not shown). To investigate the importance of type I IFNs on the production of IL-12p70 in our system, co-infections in the presence of neutralizing antibodies against IFN-α, IFN-β and the type I IFN receptor were performed. As the promoter of IL-12p35 was reported to contain an interferon-stimulated response element-1 (ISRE-1) (Goriely *et al*., 2006), quantitative real-time PCR was used to compare the transcriptional induction of both IL-12p70 subunits. Analysis of mRNA levels of IL-12p40 (Fig. 8B) and IL-12p35 (Fig. 8C) revealed that the induction of IL-12p35 was almost completely abrogated in the presence of the neutralizing antibodies, while the transcription of the p40 subunit remained unchanged. Additionally, an inhibitor of IRF-3-dependent gene transcription (Johnson *et al*., 2011) diminished the IL-12p70 secretion after bacterial infection or viral/bacterial co-infection (Fig. S7A) showing that IRF-3 plays a large role in the production of IL-12p70 in this co-infection system. In addition, the IRF-3 inhibitor also led to slightly decreased levels of IL-6 (data not shown); however, IL-12p40 levels remained unchanged (Fig. S7B). These results show that IAV-induced type I IFNs were largely responsible for the observed priming of MDDCs and that this effect enhanced the transcription of the p35 subunit of IL-12p70 in response to a subsequent infection with SP.

## Discussion

Co-infection with multiple pathogens can result in increased severity of disease symptoms. Many mechanisms involved in the cross-talk between innate receptors which are involved in poly-microbial diseases have not been fully characterized. In the present study, we show that co-infection of human MDDCs with viable IAV and SP enhance the induction and secretion of IL-12p70, as well as IL-6. The same cytokines were shown to be elevated in infected individuals during the influenza pandemic in 2009 where expression of IL-12p70, IL-6 and IL-15 correlated with severe clinical outcome (Bermejo-Martin *et al*., 2009), although co-infection with SP was not confirmed in that study.

The priming of MDDCs to promote cytokine induction in our co-infection system was dependent on virus replication, as heat-inactivated IAV failed to cause the effect. It is likely that this difference is due to lack of synthesis and/or amplification of pathogen-associated molecular patterns (PAMPs), for example RNA replication intermediates, by the heat-inactivated virus. PAMPs are recognized by receptors of the innate immune system and thereby trigger immune responses in infected cells, whereas in cells stimulated with heat-inactivated virus these PAMPs are absent or present in amounts too low to cause the observed effects. This hypothesis is supported by the fact that the priming of MDDCs to produce IL-12p70, as well as IL-6, could be mimicked by various TLR agonists and type I IFN in combination with bacterial infection, showing that it is not the virus infection itself but the cellular response to the infection that is responsible for the increased cytokine production. Similarly, we also showed that stimulation of IAV-infected MDDCs with heat-killed SP at late time points of viral infection does not lead to enhanced cytokine secretion (Wu *et al*., 2011). In our system, killed bacteria were very poor inducers of IL-12p70 compared with viable bacteria at the same moi. However, we also found that stimulation with TLR ligands or infection with IAV for longer time periods strongly decreased the secretion of IL-12p70 after infection with SP. We demonstrated that uptake of SP and endosome acidification is required for the induction of IL-12p70 since this cytokine could not detected if phagocytosis or acidification was blocked with cytochalasin D or ammonium chloride respectively. Attachment of bacteria to the cell surface was insufficient for the induction of IL-12p70 since analysis of intracellular cytokines revealed that only a small percentage of cells produced IL-12p70, despite the fact that the majority of the MDDCs exposed to SP were associated with bacteria. In addition, immunofluorescence analysis showed a minor population of cells with a cytoplasmic stain for SP. Taken together, these data indicate that MDDCs need to engulf and digest SP in order to stimulate the production of IL-12p70. This may explain why IAV-infected or TLR-stimulated MDDCs at later time points are non-responsive in terms of IL-12p70 induction since we found that bacterial uptake inversely correlated over time with the amount of applied virus or TLR agonists. MDDCs infected with IAV or stimulated with TLR agonists for 24 h almost completely abrogated uptake of bacteria. Contrary to this, no increase of bacterial uptake was observed after infection with IAV or stimulation with TLR agonists at conditions where enhanced cytokine secretion was observed. This indicates that the induction is instead due to enhanced responsiveness of the MDDCs rather than a stronger stimulation augmented by uptake of SP. In addition, applying higher moi of SP diminished the induction of IL-12p70, suggesting that the pathways involved are tightly regulated and excessive stimulation may result in negative regulation. It remains to be investigated why viable bacteria were superior in the induction of cytokines. Again, we speculate that the difference is likely mediated by a PAMP, which is recognized by the MDDCs but is not present to the same extent in killed compared with viable bacteria. A recent study showed that prokaryotic mRNA is a PAMP that can be detected by the immune system and responses to this PAMP are dependent on the viability of the bacteria (Sander *et al*., 2011). Although this was shown for Gram-negative bacteria it seems plausible that a similar mechanism is responsible for the lack of cytokine induction that we observed after application of killed SP. Other recent reports showed that single-stranded RNA of Gram-positive bacteria is recognized as a PAMP in a TLR-dependent manner, which is also dependent on phagocytosis of the bacteria (Cervantes *et al*., 2011; Hidmark *et al*., 2012).

Our data indicate that co-infection of the same cell is not required for the induction of IL-12p70. Rather the IAV-infected MDDCs stimulate uninfected cells that have taken up SP to produce enhanced levels of IL-12p70 and that this likely occurs via the release of extracellular mediators. Small amounts of type I IFNs stimulate an anti-viral state in uninfected cells and allow them to enhance their response towards infection. This priming leads to an upregulation of receptors of the innate immune system, signalling molecules and transcription factors and thereby prepares the cells for a possible subsequent infection (Stewart *et al*., 1971; Osterlund *et al*., 2005; Phipps-Yonas *et al*., 2008; Kuri *et al*., 2009). Using recombinant IFN-α we could partially mimic the effects of the preceding virus infection, although supernatants of IAV-infected cells were superior in inducing IL-12p70. A similar result was also observed for the induction of IL-6. This can possibly be explained by the viral induction of IFN-β and several types of IFN-α which act in a superior way compared with recombinant IFN-α only (Fig. S8A and B). Even though all type I IFNs use the same receptor it has been shown that they bind with different affinities and even induce different sets of interferon-stimulated genes (Lavoie *et al*., 2011). Nevertheless, blocking the effects of type I IFNs with antibodies resulted in a loss of the priming of IL-12p70 induction, showing that type I IFNs mediated the priming. Importantly, in support of this we showed that an IAV-infected lung epithelial cell line was also capable of priming MDDCs and thereby promoting the induction of IL-12p70.

A similar scenario may occur *in vivo* especially since the enhanced cytokine response does not require co-infection of the same cell but could be mediated without close spatial or even temporal proximity of viral and bacterial infected cells. IAV-infected cells releasing the appropriate priming signals could stimulate neighbouring cells and thereby cause elevated cytokine responses after a subsequent pneumococcal infection. This could lead to a misguided immune response since IL-12p70 promotes Th1 responses (Macatonia *et al*., 1995) while a Th2 response would be more favourable for clearance of a pneumococcal infection (Alonso De Velasco *et al*., 1995). In contrast to our results, which show a clear effect on the p35 subunit of IL-12p70, Negishi *et al*. (2012) reported that in a murine system virus-induced activation of RIG-I-like receptors (RLR) selectively suppressed transcription of the p40 subunit of IL-12p70 and suppressed bacterial induction of Th1 and Th17 responses. In the murine system this resulted in death after administration of sublethal doses of bacteria. In our study we did not observe an effect of the co-infection on the p40 subunit of IL-12p70. Both studies clearly show a skewing of the host response during polymicrobial disease and differences between the findings may be related to a number of factors predominantly the difference in host species, since we have previously reported a distinct difference in the production of IL-12p70 in response to pneumococci in murine and human DCs (Littmann *et al*., 2009). In support of this, type I IFNs have recently been shown to enhance cross-presentation by human DCs, which is also favoured by Th1 (Spadaro *et al*., 2012). IL-6 could promote Th17 responses and elevated levels of this cytokine are also expected to result in enhanced inflammation. As Th1 and Th17 hypercytokinaemia was found to be associated with severe outcome of IAV infection (Bermejo-Martin *et al*., 2009), the cytokine pattern we observed could contribute to that unfortunate immune response.

We used IAV and SP for our co-infection studies but since we could largely mimic the effect by stimulating the MDDCs with TLR agonists these findings may not be restricted to these specific pathogens but may also be applicable to other viral and bacterial co-infections, especially as the induction of type I IFNs is a hallmark of virus infections. It remains to be investigated how cytokines like IL-12p70 and IL-6, which are induced and regulated by multiple pathways, are similarly affected by type I IFN priming. Likely, elements of the induction pathways are represented by interferon-stimulated genes since priming with type I IFNs enhanced the expression of both cytokines after SP infection. In addition, an alternative induction route for IL-6, involving IL-1 receptor signalling was recently described (Cahill and Rogers, 2008). As IAV infection leads to secretion of low amounts of IL-1β (Ichinohe *et al*., 2010), it could explain why IL-6 is more strongly induced than other cytokines, which in general depend on the same transcription factors, e.g. NFκB in their classical induction pathways. Understanding the mechanism behind the priming of these cytokines is important as such knowledge may provide new therapeutic avenues for human co-infections with viruses and bacteria.

## Experimental procedures

### Virus and bacteria

The X31 strain of IAV (Kilbourne, 1969) was propagated in chicken eggs. The virus stocks were then purified and concentrated on sucrose gradient (Virapur) and virus titres were determined by performing Avicel™ (FMC Bioploymer) plaque assays on Madine Darby canine kidney (MDCK) cells as described (Matrosovich *et al*., 2006). Infection rates of MDDCs were determined by immunofluorescence staining (see *Immunofluorescence analysis*). For heat inactivation, IAV stocks were incubated for 10 min at 56°C. Virus inactivation was confirmed by incubation of MDCK cells with the heat-inactivated stocks and the infection status of the cells was monitored by virus-specific immunofluorescence staining (no infected cells were found). Unencapsulated SP of serotype 4 (TIGR4) (Fernebro *et al*., 2004) was grown overnight on blood agar plates at 37°C and 5% CO_2_. Colonies were inoculated into C + Y medium and grown to OD_620_ = 0.5. Dilutions were made to obtain the desired concentration of bacteria and viable counting was performed to retrospectively confirm bacterial numbers. For gentamicin killing, SP was grown as described and then incubated with gentamicin (100 μg ml^−1^) for 1 h at 37°C and 5% CO_2_. Killing of the bacteria was confirmed by plating on blood agar plates. If not otherwise stated a moi of 0.5 for IAV and moi of 1 for SP was used to infect the MDDCs.

### Preparation of MDDCs

Monocytes were purified from buffy coats of healthy donors (Karolinska University Hospital) using a RosetteSep™ monocyte purification kit (Stem Cell Technologies) and Ficoll-Hypaque Plus (Amersham Biosciences) gradient centrifugation. Human MDDCs were seeded at 0.5–1 × 10^6^ cells ml^−1^ in R10 (RPMI 1640, 2 mM l-glutamine, 10% FCS) supplemented with GM-CSF (40 ng ml^−1^) and IL-4 (40 ng ml^−1^) for 6 days. Cells were given fresh media and cytokines at a ratio of 1:1 on day 4, and cultured until day 6. The human MDDC phenotype was assessed by examination of CD11c and CD1a expression using allophycocyanin (APC)-conjugated mouse anti-human CD11c and fluorescein isothiocyanate (FITC)-conjugated mouse anti-human CD1a (BD Pharmingen). MDDCs used in these experiments were above 93% CD1a/CD11c^+^.

### Co-infection set-up

MDDCs were seeded in 96-well plates (1 × 10^5^ per well) and exposed to IAV or heat-inactivated IAV for 1 h in serum-free conditions and for a subsequent 3 h in the presence of serum. Cells were pelleted, medium was removed and SP was added in fresh R10 medium. After 2 h incubation gentamicin (100 μg ml^−1^) was added to kill extracellular bacteria and maintained in the cell culture until the end of the experiment. Experiments were performed using three replicate wells for each condition.

### Neutralization of type I IFNs

MDDCs were seeded and infected in serum-free conditions for 1 h as described above. Cells were pelleted and medium was replaced with R10 medium containing antibodies against human IFN-α (No. 31101-1, PBL Interferon source), IFN-β (No. 31410-1, PBL Interferon source) and IFNAR (MMHAR-2, PBL Interferon source) at concentrations of 5000 U ml^−1^, 2000 U ml^−1^ and 20 μg ml^−1^ respectively. As a control, cells were incubated with unspecific IgG of the corresponding species at equivalent concentrations. The antibodies were maintained in the medium until the end of the experiment. Bacteria and gentamicin were added as described above.

### Quantification of bacterial uptake

MDDCs were infected with IAV or stimulated with TLR agonists for 4 h or 24 h before SP at moi of 1 was added. Control cells were subjected to the same conditions in the absence of virus. After 2 h of infection with SP, gentamicin (400 μg ml^−1^) was added to cultures for 1 h to kill extracellular bacteria. Cells were then washed two times with PBS, lysed with 0.1% saponin (Sigma) in PBS for 5 min and the numbers of viable internalized bacteria were enumerated by viable count on blood agar plates. For quantification of SP at later time points than 3 h post infection, 100 μg ml^−1^ gentamicin was added to cultures 2 h after infection and maintained in the cell culture until the cells were lysed and plated as described above. Experiments were performed using four replicate wells for each condition. In order to account for donor variation, the uptake of SP in the absence of any co-treatment was adjusted to 1.

### Inhibitors and reagents

Cytochalasin D (0.5 μM, Sigma) was applied to block phagocytosis of MDDCs and NH_4_Cl (5 mM) was used to inhibit endosome acidification. Both treatments were applied 15 min prior to infection with SP. Gö6976 (Sigma-Aldrich) was applied at the indicated concentrations 2 h before the bacteria were added to the cells.

### Quantification of cytokines

For cytokine assessment culture supernatants were harvested 24 h after infection and used directly for measurement of IL-12p70 by ELISA or frozen at −20°C for measurement of TNFα, IL-1β, IFN-β, IL-12p40, IL-23, IL-6, IL-8 and IL-10. Concentrations of IL-12p70, IL-12p40, IL-6, IL-8, IL-23 and TNFα were measured using OptEIA™ ELISA kits (BD Biosciences). IL-10 and IL-1β were measured using the Ready Set Go! ELISAs kits from eBioscience and IFN-β concentrations were determined using the VeriKine-HS™ IFN-β-Kit (PBL interferon source).

### Intracellular cytokine staining

MDDCs were sequentially infected with IAV and SP as described above. After co-infection, 1 μg ml^−1^ brefeldin A (BD Biosciences) or 2 μM monensin (eBioscience) were added to the cells for 14 h to allow accumulation of intracellular IL-12p70, IL-12p40, TNFα or IL-6 respectively. MDDCs were washed and fixed with 3.7% paraformaldehyde (Sigma) for 10 min at room temperature, permeabilized with 0.1% saponin (Sigma) and stained with antibodies against CD14 (clone MΦP9, BD Biosciences), IL-12p70 (clone 20C2, BD Pharmingen), IL-12p40 (eBioscience), TNFα (BD Pharmingen), IL-6 (eBioscience) and influenza nucleoprotein (clone A3, Chemicon) for 20 min at 4°C. Cells were washed in permeabilization buffer and analysed by flow cytometry using a FACSCalibur (BD).

### Immunofluorescence analysis

MDDCs were seeded on Lab-Tech™ chamber slides (Nunc) and sequentially infected with IAV and SP as described above. At 4 h after infection with SP the cells were washed with PBS, fixed and permeabilized with methanol/acetone (70/30 v/v) for 10 min at room temperature and stained for IAV nucleoprotein (clone A3, Chemicon) and SP (Novus Biologicals). After incubation for 1 h at room temperature the slides were washed with PBS, then treated with 1 μg ml^−1^ Hoechst 33258 DNA staining reagent (Invitrogen) and with the secondary antibodies Cy3-conjugated donkey anti-mouse IgG (Jackson Immunoresearch) and Cy2-conjugated donkey anti-rabbit IgG (Jackson Immunoresearch) at a dilution of 1:1000 and 1:200 respectively. Slides were then washed again and mounted with vinol mounting medium. Stained cell samples were examined by using a Leitz DM RB fluorescence microscope with a Hamamatsu cooled charge-coupled-device C4880 camera. Images were processed and compiled using Adobe Photoshop.

### Supernatant transfer experiments

MDDCs were infected with IAV as described above. After incubation for 24 h the supernatants were harvested, cleared from cell debris by centrifugation and stored at −20°C. The presence of infectious virus particles was excluded by incubation of fresh MDDCs with the supernatants and subsequent immunofluorescence analysis for viral nucleoprotein and detection of viral M1-mRNA in real-time PCR. MDDCs in R10 medium were seeded in 96-well plates (1 × 10^5^ per well) and incubated with the supernatants of mock or IAV-infected cells (one-fourth of total volume) for 4 h prior to infection with SP. The cells were pelleted, medium was removed and SP was added together with supernatant of mock or IAV-infected cells (one-fourth of total volume). After 2 h incubation, gentamicin (100 μg ml^−1^) was added to kill extracellular bacteria and maintained in the cell culture for up to 24 h. Cytokine levels in supernatants were then determined by ELISA.

### A549/MDDC co-culture experiments

Human epithelial lung cells (A549) were seeded in 48-well plates to ∼ 70–80% confluency and infected with IAV at moi 1 for 1 h under serum-free conditions. The cells were then washed three times to remove unbound virus and further incubated in R10 medium for 3 h. After this, 1 × 10^5^ MDDCs with or without SP were added to the A549 cells and incubated for 2 h before gentamicin (100 μg ml^−1^) was added to kill extracellular bacteria and maintained in the cell culture until the end of the experiment. The cells were incubated for further 16 h before the concentration of IL-12p70 in the supernatants was determined.

### Real-time PCR

Total RNA of MDDCs was isolated by using the RNeasy kit (Qiagen). Reverse transcription was performed by using the Quantitect RT kit (Qiagen) and generated cDNA was used for subsequent real-time PCR mRNA levels of human γ-actin (QuantiTect primers QT00996415) and viral M1 (Kash *et al*., 2011) were detected using a QuantiTect SYBR Green PCR kit (Qiagen) and a QuantiTect Probe PCR kit (Qiagen) respectively. Real-time PCR was performed applying an ABI 7500 PCR system. Reactions were performed in duplicates and the Ct values were normalized to γ-actin. ΔΔCt method was applied to calculate the ‘fold’ induction relative to uninfected control cells.

### Assessment of cell viability

The influence of the different treatments and infections on the viability of the MDDCs was monitored using a cytotoxicity detection kit (Roche). Briefly, cell culture supernatants were collected and assayed for the presence of lactate dehydrogenase. The assay was performed according to the manufacturer’s instructions.

### Statistical analysis

Graphs show average ± SEM values from independent experiments of MDDCs from different donors, if not otherwise specified. Statistical significances were tested using paired Student’s *t*-test in Graphpad Prism 5.0. Values of *P* < 0.05 were considered statistically significant and are represented as *P < 0.05; **P < 0.001; ***P < 0.0001; ns, not statistically significant; nd, not detected.

## Supplementary material

Fig S1 Preceding influenza infection leads to elevated levels of IL-6 after co-infection with SP. MDDCs were infected with IAV for 4 h before SP was added. The cells were incubated for a further 18 h before the concentration of IL-6 in the supernatants was measured by ELISA. Different infection conditions were tested for their potential to induce an elevated cytokine response. A. IAV/heat-inactivated (HI) IAV moi 0.5 and SP moi 1. B. IAV/HI-IAV moi 0.5 and viable or gentamicin-killed SP moi 1. C. Increasing moi of IAV and SP moi 1. D. Increasing moi of SP. The graphs show cytokine concentrations derived from cells of one representative donor out of (A) six, (B) two, (C) seven, (D) three different donors.

Fig S2 Clearance of phagocytosed SP after IAV infection and bacterial uptake after infection with Semliki Forest virus (SFV). A. MDDCs were infected with IAV for 4 h before SP was added. At indicated time points the cells were lysed and viable, intracellular SP was enumerated. The graph shows mean ± SEM of independent experiments with three different donors. B. MDDCs were infected with SFV at different moi for the indicated time points before bacterial uptake was monitored. Values represent mean ± SEM of independent experiments with a minimum of three different donors. Statistical analysis was performed using paired Student’s t-test. (****P* < 0.001).

Fig S3 Increased secretion of IL-6 can be stimulated by a combination of different TLR agonists and SP. Different doses of a TLR3 (A), TLR7/8 (B) or TLR4 agonist (C) were applied for 4 h before SP was added. The cells were incubated for another 18 h and concentration of IL-6 in supernatants was determined by ELISA. The graphs show cytokine concentrations derived from cells of one representative donor out of (A, B) three, (C) two different donors.

Fig S4 IAV infection does not enhance uptake and digestion of SP. MDDCs were seeded on glass slides and infected with SP only or sequentially infected as described before. The cells were fixed with paraformaldehyde 4 h after addition of SP and stained with specific antibodies for SP and Hoechst DNA stain. Four hundred cells per donor were examined and the percentage of cells with cytoplasmic stain for SP was determined. The numbers show the average frequency for independent experiments with three donors ± SEM. Statistical analysis was performed using paired Student’s *t*-test.

Fig S5 Supernatants of IAV-infected MDDCs prime non-infected cells to launch a stronger IL-6 response towards SP. A. MDDCs were infected with IAV or treated with supernatants of mock-(SN mock) or IAV-infected MDDCs (SN IAV) and incubated for 4 h. The cells were then infected with SP and further incubated for 18 h before the concentration of IL-6 in the supernatants was measured by ELISA. The graph shows results of one representative experiment out of five with different donors. No infectious IAV particles in supernatant of infected MDDCs detectable. B. MDDCs were incubated with supernatants of uninfected (top row), IAV infected (middle) or supernatants of IAV-infected MDDCs which were spiked with IAV stocks (bottom row) and incubated for 16 h. The cells were then fixed with paraformaldehyde and stained for the presence of IAV nucleoprotein. C. MDDCs were infected with IAV, or incubated with supernatants of uninfected or IAV-infected MDDCs. After 16 h of incubation the cells were lysed and total RNA was isolated and assayed for the presence of IAV M1 mRNA. Ct values were normalized against γ-actin and the relative induction of genes was calculated using the ……Ct method. Values represent mean ± SD of two independent experiments with different donors.

Fig S6 IL-6 secretion after IFN-priming and infection with SP. MDDCs were incubated with different concentrations of recombinant IFN-*α* 4 h prior to infection with SP. The cells were further incubated for 18 h before the concentration of IL-6 in the supernatants was measured by ELISA. The graph shows mean ± SEM from three independent experiments with different donors.
