# Supplementary material for: Influenza A virus-mediated priming enhances cytokine secretion by human dendritic cells infected with Streptococcus pneumoniae
Source: Cell Microbiol. 2013 Mar 14;15(8):1385–400. doi: 10.1111/cmi.12122 (PMC3798092; doi:10.1111/cmi.12122)
Supplement: Supplementary file 1 [file cmi0015-1385-sd1.pdf]

**Appendix S1** *Implications of multiple sources of heterogeneity for control success*

In the main paper we explore the implications for control of key hosts that exhibit only one form of asymmetry (either super-abundant, super-infected or super-shedder). In reality however, host species are likely to show asymmetry in several of these aspects (see empirical data in main paper). Here we explore the implications of such mixed mechanisms for control success. Specifically, we assume that species *i* is a key host in the sense that it contributes a significant proportion, *T*, to overall transmission, but it does so through the equal combination of two sources of asymmetry. That is, host species *i* is a key host either due to a combination of being super-abundant and super-infected, super-abundant and super-shedding or super-infected and super-shedding; in all cases both mechanisms are assumed to contribute equally to the overall degree of asymmetry of that host (see below). As in the main paper, for each key host scenario we explored the effect of control that removes a certain number of individuals of species *i* ($C_i$) under two control possibilities: (1) Untargeted control, where $C_i$ individuals are removed regardless of infection status and (2) Targeted control, where only infected individuals are removed. Again, we quantified the impact of control as the proportion of the parasite's initial infectious pool remaining after either targeted ($\xi^T$) or untargeted ($\xi^U$) control (Eqns 2 and 3, main paper).

*Assuming mechanisms of asymmetry are equal*

From the main paper we know the overall contribution of host species *i* to parasite transmission is given by:

$$\pi_i = \frac{\theta_i^A \theta_i^I \theta_i^S}{N} = T$$

If we consider the case where species *i* is a key host through two mechanisms (e.g. $\theta_i^x$ and $\theta_i^y$ >> 1, $\theta_i^z = 1$, where *x*, *y* and *z* are each one of the three mechanisms asymmetry), this becomes:

$\frac{\theta_i^x \theta_i^y}{N} = T$ , or $\theta_i^x = \frac{NT}{\theta_i^y}$.

Hence, multiple combinations of $\theta_i^x$ and $\theta_i^y$ (any pair of mechanisms) will allow host species *i* to contribute a proportion *T* to overall parasite transmission. For this reason, in what follows we assume both mechanisms contribute equally to transmission, such that:

$$\theta_i^x = \theta_i^y = \sqrt{NT} \qquad \text{Eq S1,}$$

which provides a single, unique value for the magnitude of each asymmetry.

*Super-abundant and super-infected host*

Here $\theta_i^S$=1, so $\lambda_i = \frac{\sum_j \mathrm{H}_j \mathrm{p}_j \lambda_j}{\sum_j \mathrm{H}_j \mathrm{p}_j}$. Furthermore, since $\theta_i^I = \sqrt{NT}$ (from Eq S1), $\mathrm{p}_i = \frac{\sqrt{NT} \sum_j \mathrm{H}_j \mathrm{p}_j}{\sum_j \mathrm{H}_j}$.

Hence, from Eqs 2 and 3 in the main paper:

$$\xi^T = 1 - \frac{C_i}{\sum_j H_j p_j}$$

and

$$\xi^U = 1 - \frac{C_i \sqrt{NT}}{\sum_j H_j}.$$

*Super-abundant and super-shedding host*

Here $\theta_i^I$=1, so $\mathrm{p}_i = \frac{\sum_j \mathrm{H}_j \mathrm{p}_j}{\sum_j \mathrm{H}_j}$. Furthermore, since $\theta_i^S = \sqrt{NT}$ (from Eq S1), $\lambda_i = \frac{\sqrt{NT} \sum_j \mathrm{H}_j \mathrm{p}_j \lambda_j}{\sum_j \mathrm{H}_j \mathrm{p}_j}$.

Hence

$$\xi^T = 1 - \frac{C_i \sqrt{NT}}{\sum_j H_j p_j}$$

and

$$\xi^U = 1 - \frac{C_i \sqrt{NT}}{\sum_j H_j}.$$

*Super-infected and super-shedding host*

Here $\theta_i^S = \sqrt{NT}$, so $\lambda_i = \frac{\sqrt{NT}\sum_j \mathrm{H}_j \mathrm{p}_j \lambda_j}{\sum_j \mathrm{H}_j \mathrm{p}_j}$, and so:

$$\xi^T = 1 - \frac{C_i\sqrt{NT}}{\sum_j H_j p_j}$$

Furthermore, since $\theta_i^I \theta_i^S = NT$, $\mathrm{p}_i \lambda_i = \frac{NT\sum_j \mathrm{H}_j \mathrm{p}_j \lambda_j}{\sum_j \mathrm{H}_j}$. Hence:

$$\xi^U = 1 - \frac{C_i NT}{\sum_j H_j}.$$

## Results

Figure S1 illustrates the effects of (a) untargeted and (b) targeted control under these various scenarios (which can be compared to Fig. 1, which assumed only single mechanisms of asymmetry). In general the efficacy of a given control effort under multiple sources of heterogeneity (Fig. S1) lies between the two extremes of the relevant single-sources of heterogeneity (Fig. 1). For example, untargeted control is more effective (fewer individuals need to be treated) for a mixed super-abundant and super-infected key host (Fig. S1a, purple line) than an equivalent purely super-abundant key host (Fig. 1a, black line). However, the same mixed key host is harder to control than the equivalent purely super-infected key host (Fig. 1a, red line). Specifically, moving from one extreme single mechanism (e.g. a pure super-abundant host) to the equal mixed case (e.g., an equally super-abundant and super-infected host) to the other extreme single mechanism (e.g. a pure super-infected host) alters the efficacy of control by a factor $\sqrt{NT}$ each step (Table S1).
