# Supplementary material for: Influenza A virus-mediated priming enhances cytokine secretion by human dendritic cells infected with Streptococcus pneumoniae
Source: Cell Microbiol. 2013 Mar 14;15(8):1385–400. doi: 10.1111/cmi.12122 (PMC3798092; doi:10.1111/cmi.12122)
Supplement: Supplementary file 2 [file cmi0015-1385-sd2.pdf]

Appendix S2 *Study sites, host and parasite data*

Study sites in Virginia included Center Forest (CF, Montgomery County) and Mountain Lake Biological Station (MLBS, Pembroke County). Grids in Tennessee followed a gradient from 454 to 1960 meters on Sugarland Mountain in the Great Smoky Mountains National Park (GSM, Sevier County). The 7 GSM grids were grouped into low (≤ 638 metres) and high elevation (≥ 785 metres) sites based on changes in the identity of the dominant mouse species in the host community (*P. leucopus* in low elevation; *P. maniculatus* in high elevation grids, Fig. 2). Sites in New York and Connecticut were at the Cary Institute of Ecosystem Studies (IES, Dutchess County) and Great Mountain Forest (GMF, Litchfield County), respectively.

At each site, trapping grids were laid out in 10x10, 8x8, or 6x6 arrays, with 15 meters between traps. Grid sizes varied because we used both established and new trapping sites, and were limited by space. Sherman live traps were opened, baited with crimped oats after 1600 hours, and checked the following morning between 0700 and 1100 hours. We trapped 2-3 consecutive nights at each site from June – August 2003. On new trapping sites, hair was trimmed from the posterior, dorsal region of all captured animals for identification of recaptured individuals. Ear tags were used to identify individuals on established grids. Animals were released at the site of capture. Traps were washed with a 5% Lysol solution between trapping sessions to avoid faecal contamination across sites and species.

Faeces were collected from traps containing animals, weighed and preserved in 10% formalin. We used faecal flotation in a saturated NaCl solution followed by light microscopy to diagnose parasite infection and generate estimates of parasite egg/oocyst shedding per gram of faeces (Pritchard & Kruse 1982). We were unable to identify all parasite eggs to species, but described pseudo-species by comparing egg morphology and measurements to previous accounts

of intestinal parasites in wild rodents (Doran 1954a, b; Levine & Ivens 1965; Forster 1984; Pedersen 2005).

Appendix S2 References

1.

Doran D.J. (1954a). A catalogue of the protozoa and helminths of North American rodents. II. Cestoda. *Am. Midl. Nat.*, 53, 469-480.

2.

Doran D.J. (1954b). A catalogue of the protozoa and helminths of North American rodents. III. Nematoda. *Am. Midl. Nat.*, 53, 162-175.

3.

Forster S.L. (1984). Ecology of helminths in Peromyscus. MS Thesis: *Department of Biology*. University of Virginia Charlottesville, Virginia.

4.

Levine N. & Ivens V. (1965). *Coccidian Parasites (Protozoa, Sporozoa) of Rodents*. University of Illinois Press, Urbana, Illinois, USA.

5.

Pedersen A.B. (2005). Intestinal parasites, acorn masts and population dynamics of *Peromyscus*. PhD Thesis: *Department of Biology*. University of Virginia Charlottesville, Virginia, p. 246.

6.

Pritchard M.H. & Kruse G.O.W. (1982). *The collection and preservation of animal parasites*. University of Nebraska Press, Lincoln, Nebraska, USA.
