# Supplementary material for: Influenza A virus-mediated priming enhances cytokine secretion by human dendritic cells infected with Streptococcus pneumoniae
Source: Cell Microbiol. 2013 Mar 14;15(8):1385–400. doi: 10.1111/cmi.12122 (PMC3798092; doi:10.1111/cmi.12122)

Figure S1 *Efficacy of control by untargeted (a) and targeted (b) removal with respect to infection status of a key host species with multiple sources of heterogeneity*. In each scenario, the host species is assumed to be responsible for 80% of the total contribution to the parasite's infectious pool (T=0.8). The dashed line represents the maximum reduction in transmission possible by treating only the key host (i.e., the proportion of transmission that is due to the other non-host species). For visualization, the gold lines (super-abundant and super shedder key hosts) are offset to avoid overlap with super-abundant, super-infected key hosts (panel a) and super-infected, super-shedder key hosts (panel b).

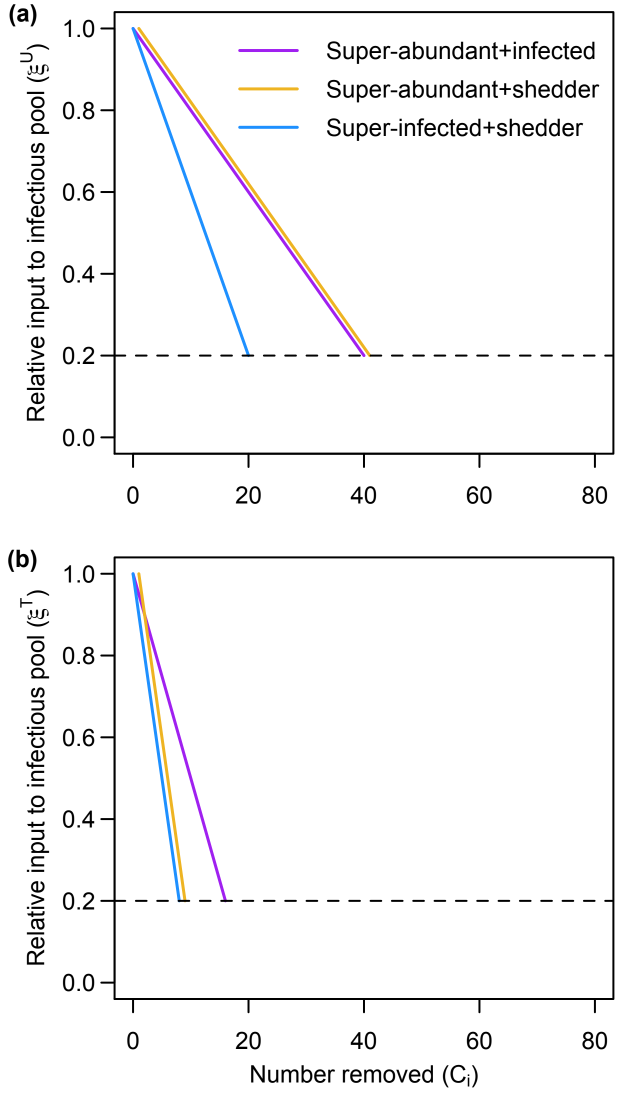

Supplement: Fig S1 — Preceding influenza infection leads to elevated levels of IL-6 after co-infection with SP. MDDCs were infected with IAV for 4 h before SP was added. The cells were incubated for a further 18 h before the concentration of IL-6 in the supernatants was measured by ELISA. Different infection conditions were tested for their potential to induce an elevated cytokine response. A. IAV/heat-inactivated (HI) IAV moi 0.5 and SP moi 1. B. IAV/HI-IAV moi 0.5 and viable or gentamicin-killed SP moi 1. C. Increasing moi of IAV and SP moi 1. D. Increasing moi of SP. The graphs show cytokine concentrations derived from cells of one representative donor out of (A) six, (B) two, (C) seven, (D) three different donors. [file cmi0015-1385-sd3.pdf]
