# Supplementary material for: Influenza A virus-mediated priming enhances cytokine secretion by human dendritic cells infected with Streptococcus pneumoniae
Source: Cell Microbiol. 2013 Mar 14;15(8):1385–400. doi: 10.1111/cmi.12122 (PMC3798092; doi:10.1111/cmi.12122)
Supplement: Fig S2 — Clearance of phagocytosed SP after IAV infection and bacterial uptake after infection with Semliki Forest virus (SFV). A. MDDCs were infected with IAV for 4 h before SP was added. At indicated time points the cells were lysed and viable, intracellular SP was enumerated. The graph shows mean ± SEM of independent experiments with three different donors. B. MDDCs were infected with SFV at different moi for the indicated time points before bacterial uptake was monitored. Values represent mean ± SEM of independent experiments with a minimum of three different donors. Statistical analysis was performed using paired Student’s t-test. (***P < 0.001). [file cmi0015-1385-sd4.doc]

**Figure S2** *Scatterplots showing relationships among the three forms of asymmetry*. Each point represents asymmetry values for a particular host species-parasite species comination, with points coloured according to host species.
