# Supplementary material for: Influenza A virus-mediated priming enhances cytokine secretion by human dendritic cells infected with Streptococcus pneumoniae
Source: Cell Microbiol. 2013 Mar 14;15(8):1385–400. doi: 10.1111/cmi.12122 (PMC3798092; doi:10.1111/cmi.12122)
Supplement: Fig S3 — Increased secretion of IL-6 can be stimulated by a combination of different TLR agonists and SP. Different doses of a TLR3 (A), TLR7/8 (B) or TLR4 agonist (C) were applied for 4 h before SP was added. The cells were incubated for another 18 h and concentration of IL-6 in supernatants was determined by ELISA. The graphs show cytokine concentrations derived from cells of one representative donor out of (A, B) three, (C) two different donors. [file cmi0015-1385-sd5.doc]

**Figure S3** *Magnitude of abundance and shedding asymmetry for 11 gastrointestinal parasites*. Values of θA > 1 (horizontal dashed line) indicate high abundance relative to the community average. Values of θS > 1 (vertical dashed line) indicate high shedding of parasite infectious stages relative to the community average. Circle sizes are coloured according to host species and sized proportionally to each species *i* contribution to the infectious pool (π_i_).
