# Supplementary material for: Influenza A virus-mediated priming enhances cytokine secretion by human dendritic cells infected with Streptococcus pneumoniae
Source: Cell Microbiol. 2013 Mar 14;15(8):1385–400. doi: 10.1111/cmi.12122 (PMC3798092; doi:10.1111/cmi.12122)
Supplement: Fig S4 — IAV infection does not enhance uptake and digestion of SP. MDDCs were seeded on glass slides and infected with SP only or sequentially infected as described before. The cells were fixed with paraformaldehyde 4 h after addition of SP and stained with specific antibodies for SP and Hoechst DNA stain. Four hundred cells per donor were examined and the percentage of cells with cytoplasmic stain for SP was determined. The numbers show the average frequency for independent experiments with three donors ± SEM. Statistical analysis was performed using paired Student’s t-test. [file cmi0015-1385-sd6.doc]

**Figure S4** *Magnitude of abundance and infection asymmetry for 11 gastrointestinal parasites*. Values of θA > 1 (horizontal dashed line) indicate high abundance relative to the community average. Values of θI > 1 (vertical dashed line) indicate high parasite infection relative to the community average. Circle sizes are coloured according to host species and sized proportionally to each species *i* contribution to the infectious pool (π_i_).
