# Supplementary material for: Influenza A virus-mediated priming enhances cytokine secretion by human dendritic cells infected with Streptococcus pneumoniae
Source: Cell Microbiol. 2013 Mar 14;15(8):1385–400. doi: 10.1111/cmi.12122 (PMC3798092; doi:10.1111/cmi.12122)
Supplement: Fig S5 — Supernatants of IAV-infected MDDCs prime non-infected cells to launch a stronger IL-6 response towards SP. A. MDDCs were infected with IAV or treated with supernatants of mock-(SN mock) or IAV-infected MDDCs (SN IAV) and incubated for 4 h. The cells were then infected with SP and further incubated for 18 h before the concentration of IL-6 in the supernatants was measured by ELISA. The graph shows results of one representative experiment out of five with different donors. No infectious IAV particles in supernatant of infected MDDCs detectable. B. MDDCs were incubated with supernatants of uninfected (top row), IAV infected (middle) or supernatants of IAV-infected MDDCs which were spiked with IAV stocks (bottom row) and incubated for 16 h. The cells were then fixed with paraformaldehyde and stained for the presence of IAV nucleoprotein. C. MDDCs were infected with IAV, or incubated with supernatants of uninfected or IAV-infected MDDCs. After 16 h of incubation the cells were lysed and total RNA was isolated and assayed for the presence of IAV M1 mRNA. Ct values were normalized against γ-actin and the relative induction of genes was calculated using the ……Ct method. Values represent mean ± SD of two independent experiments with different donors. [file cmi0015-1385-sd7.doc]

**Figure S5** *Magnitude of shedding and infection asymmetry for 11 gastrointestinal parasites.* Values of θS > 1 (horizontal dashed line) indicate high shedding of parasite infectious stages relative to the community average. Values of θI > 1 (vertical dashed line) indicate high parasite infection relative to the community average. Circle sizes are coloured according to host species and sized proportionally to each species *i* contribution to the infectious pool (π_i_).
