# Supplementary material for: Influenza A virus-mediated priming enhances cytokine secretion by human dendritic cells infected with Streptococcus pneumoniae
Source: Cell Microbiol. 2013 Mar 14;15(8):1385–400. doi: 10.1111/cmi.12122 (PMC3798092; doi:10.1111/cmi.12122)
Supplement: Fig S6 — IL-6 secretion after IFN-priming and infection with SP. MDDCs were incubated with different concentrations of recombinant IFN-α 4 h prior to infection with SP. The cells were further incubated for 18 h before the concentration of IL-6 in the supernatants was measured by ELISA. The graph shows mean ± SEM from three independent experiments with different donors. [file cmi0015-1385-sd8.doc]

**Figure S6** *Predicted efficacy of three hypothetical control strategies for 11 parasites after accounting for unknown variation in capture probabilities across host species*. Each panel shows the expected reduction in the infectious pool size by random removal of individuals regardless of host species (green) and by targeted (blue) and untargeted (red) removal of the most influential host species (shown in the title of each panel). The solid lines show the mean of the efficacy of each control strategy from 100 iterations of randomly assigning capture probabilities between 10 and 60% to each host species, and the shaded areas show the upper and lower bounds (1 standard deviation) of those 100 iterations.
