# Supplementary material for: Influenza A virus-mediated priming enhances cytokine secretion by human dendritic cells infected with Streptococcus pneumoniae
Source: Cell Microbiol. 2013 Mar 14;15(8):1385–400. doi: 10.1111/cmi.12122 (PMC3798092; doi:10.1111/cmi.12122)
Supplement: Supplementary file 9 [file cmi0015-1385-sd9.pdf]

Table S1 *Efficacy of untargeted and targeted control, as measured by the rate of reduction in parasite transmission with number of individuals of host species i treated (C_i)*

**Key host type**	**Untargeted control**	**Targeted control**
Pure super-abundant	$-\frac{C_i}{\sum_j H_j}$	$-\frac{C_i}{\sum_j H_j p_j}$
Pure super-infected	$-\frac{C_i NT}{\sum_j H_j}$	$-\frac{C_i}{\sum_j H_j p_j}$
Pure super-shedder	$-\frac{C_i NT}{\sum_j H_j}$	$-\frac{C_i NT}{\sum_j H_j p_j}$
Mixed<br>super-abundant & infected	$-\frac{C_i \sqrt{NT}}{\sum_j H_j}$	$-\frac{C_i}{\sum_j H_j p_j}$
Mixed<br>super-abundant & shedder	$-\frac{C_i \sqrt{NT}}{\sum_j H_j}$	$-\frac{C_i \sqrt{NT}}{\sum_j H_j p_j}$
Mixed<br>super-infected & shedder	$-\frac{C_i NT}{\sum_j H_j}$	$-\frac{C_i \sqrt{NT}}{\sum_j H_j p_j}$
