# Supplementary material for: Influenza A virus-mediated priming enhances cytokine secretion by human dendritic cells infected with Streptococcus pneumoniae
Source: Cell Microbiol. 2013 Mar 14;15(8):1385–400. doi: 10.1111/cmi.12122 (PMC3798092; doi:10.1111/cmi.12122)
Supplement: Supplementary file 10 [file cmi0015-1385-sd10.doc]

|  | Site^†^ | | | | | |
| --- | --- | --- | --- | --- | --- | --- |
| Parasite species | MLBS | CF | GMF | IES | GSM-L | GSM-H |
| Coccidia |  |  |  |  |  |  |
| *E. arizonensis A* | PL, PM^*^ | PL | PL, PM | PL | PL | PM |
| *E. arizonensis B* | PL, PM | PL | PL, PM | PL | PL | PM |
| *E. delicata* | PL, PM | PL | PL, PM | PL | PL | PM |
| Nematodes |  |  |  |  |  |  |
| *A. americana* | PL | PL | PL, PM, MG | PL |  |  |
| *C. americana* | PL, PM, TS | PL, TS, BB | MG | PL, TS, BB |  | PM, TS, MG |
| *Pterogodermatites A* | TS |  | PL, MG | PL | PL | PM, BB, MG |
| *Pterogodermatites B* | TS | BB | MG | PL, BB | PL | PM, BB |
| *Strongyle A* | PL | TS, BB |  | TS | PL | PM, MG |
| Cestodes |  |  |  |  |  |  |
| *Cestode A* |  | PL | PL, PM, MG | PL, BB |  |  |
| *H. dimunata* | PL, PM | PL |  | TS, BB | TS | PM, BB, MG |
| *H. citelli* | PL | BB |  | BB | PL | PM, BB |

**Table S2** *Geographic and host species distributions of parasites encountered in the study.*

^*^ Species abbreviations denote infection of at least 1 individual of that species; vacant cells indicate parasite absence that site. Abbreviations for infected host species names are as follows: PL = *Peromyscus leucopus*, PM = *Peromyscus maniculatus*, TS = *Tamias striatus*, BB = *Blarina brevicauda*; MG = *Myodes gapperi*. Data are pooled across multiple trapping grids in each site (see Figure 2).

^†^ Abbreviations for site names are as follows: MLBS = Mountain Lake Biological Station, Virginia; CF = Center Forest, Virginia; GMF = Great Mountain Forest, Connecticut; IES = Cary Institute of Ecosystem Studies; GSM-L = Great Smoky Mountains National Park (≤ 638m), Tennessee; GSM-H = Great Smoky Mountains National Park (≥ 785m), Tennessee.
